# Assessment of point-of-care quantitative serum canine pancreatic lipase testing for diagnosing acute pancreatitis in dogs

**DOI:** 10.3389/fvets.2025.1421103

**Published:** 2025-02-12

**Authors:** Pin-Chen Liu, Kendy Tzu-yun Teng, Tsia-Lu Lin, Chi-Hsuan Sung, Tsun-Li Cheng, Chi-Chung Chou

**Affiliations:** ^1^Department of Veterinary Medicine, College of Veterinary Medicine, National Chung Hsing University, Taichung, Taiwan; ^2^Veterinary Medical Teaching Hospital, College of Veterinary Medicine, National Chung Hsing University, Taichung, Taiwan; ^3^The iEGG and Animal Biotechnology Research Center, National Chung Hsing University, Taichung, Taiwan; ^4^Department and Graduate Institute of Pharmacology, National Defense Medical Center, Taipei, Taiwan

**Keywords:** acute pancreatitis, pancreatic lipase immunoreactivity, lipase, amylase, inter-class correlation coefficient, point-of-care

## Abstract

**Introduction:**

Current point-of-care testing for canine-specific pancreatic lipase (CPL) provides semi-quantitative measurements with binary results. Recently, a commercial point-of-care testing method (Vcheck CPL) that offers quantitative measurement of CPL has emerged. However, clinical studies on its value (or utility) are limited. Therefore, this study aimed to evaluate the clinical utility of this commercial point-of-care CPL in diagnosing dogs with suspected acute pancreatitis and to assess its correlation with a commercial semi-quantitative test and other clinicopathological variables.

**Methods:**

A prospective observational study included 33 dogs with suspected acute pancreatitis and 20 clinically healthy dogs. Serum Vcheck CPL and SNAP ® cPL were tested, and clinical consensus scores were determined by 5 internists. Eleven dogs with suspected acute pancreatitis underwent follow-up testing during hospitalization. The intra-class correlation coefficient (ICC) was used for statistical analysis to assess the agreement between assays and the internists’ consensus score.

**Results:**

Dogs with suspected acute pancreatitis had significantly higher serum Vcheck CPL (median: 843 μg/L, range: 77–2001, *p* < 0.0001) than healthy control dogs (median: 94 μg/L, range: 49–294). By day 3 of hospitalization, serum Vcheck CPL had significantly decreased in dogs with suspected acute pancreatitis compared to day 1. The ICC score between the clinical consensus score, Vcheck CPL, and SNAP ® cPL was 0.75, indicating good agreement. Serum Vcheck CPL concentration was significantly correlated with serum concentrations of amylase, lipase, creatinine, ALP, and CRP.

**Discussion:**

This study found good agreement between Vcheck CPL and SNAP ® cPL. This quantitative Vcheck CPL testing could serve as an adjunctive tool in diagnosing dogs with acute pancreatitis.

## 1 Introduction

Canine acute pancreatitis (AP) is recognized as the most prevalent exocrine pancreatic disorder in dogs (1). Despite its widespread occurrence, the precise pathogenesis of AP remains unclear (2). Clinical manifestations such as sudden onset of abdominal pain, anorexia, diarrhea, and vomiting are commonly observed in canines with AP (3). Epidemiologically, the incidence and mortality rates of AP vary globally, with reported mortality rates ranging from 27 to 58% (2), indicating its significant impact.

Diagnosing AP in dogs can be challenging due to the non-specific nature of clinical signs. While histopathology serves as the traditional gold standard, its utility is limited by potential oversight of focal lesions, variability in histopathological changes, and its invasive nature (4). Consequently, clinicians often rely on integrating routine clinicopathological features, ultrasound findings, and notably, specific serum canine pancreatic lipase (CPL) immunoreactivity for diagnosis (3).

Among the diagnostic tests for AP in dogs, specific pancreatic lipase immunoreactivity (PLI) has been reported as the most sensitive and specific serum marker, with sensitivity ranging between 86.5–93.6% and specificity between 66.3–77% (5–7). The activity-based serum lipase assay, 1,2-o-dilauryl-rac-glycero-3-glutaric acid-(60-methylresorufin) ester [DGGR] has also been used and reported to have high correlation with PLI (8). A commercialized quantitative ELISA known as the specific cPL (Spec cPL) was also developed (9, 10). Despite its favorable diagnostic performance, the availability of these tests is limited to certain laboratories. The relatively high costs of shipping and prolonged turnaround time impede its practical clinical use, especially in regions lacking access to commercial reference laboratories. Alternatively, a rapid point-of-care semiquantitative CPL immunoassay (SNAP ® cPL, IDEXX Laboratories Inc., Westbrook, ME, United States) was developed to expedite the diagnosis of pancreatitis. SNAP ® cPL not only demonstrates a good positive predictive value (11) but also exhibits good agreement (kappa coefficient = 0.78) with Spec cPL (10), More recently an in-clinic quantitative pancreatic lipase assay (Vcheck CPL, Bionote Co. LTD., Republic of Korea) has been developed. However, studies on its clinical application are limited.

Given the convenience of an onsite quantitative CPL test and the availability of an alternative tool for assisting with AP diagnosis, this study aimed to evaluate the correlation between semi-quantitative point-of-care SNAP ® cPL testing and commercial point-of-care quantitative Vcheck CPL testing in aiding the diagnosis of suspected AP in dogs, monitoring serum CPL during hospitalization, and examining the correlation between serum CPL levels and other clinicopathological variables.

## 2 Materials and methods

### 2.1 Animals and samples

A prospective observational study was conducted from April 2018 to April 2019 at the Veterinary Medical Teaching Hospital of National Chung Hsing University, enrolling a total of 32 client-owned dogs presenting with suspected clinical acute pancreatitis (AP). Among them, one dog presented suspected AP twice, resulting in a total of 33 data sets, referred to as *n* = 33 in the following description. Inclusion criteria comprised clinical signs such as anorexia, hyporexia, vomiting, abdominal pain, and/or diarrhea within 7 days of presentation, along with physical examination findings indicative of abdominal pain, dehydration, icterus, and/or fever. On the other hand, 20 clinically healthy client-owned dogs, with regular vaccinations, without a history of systemic disease and no recent exposure (within the last 6 months) to antimicrobials, anti-inflammatory medications, or any other drugs except for ecto- and endo-parasiticides, and displaying normal results in physical examination, CBC, and biochemistry tests, were included as healthy controls. Approval for the study was obtained from the National Chung Hsing University Institutional Animal Care and Use Committee (no. 107011), and informed consent was obtained from the owners of all participating dogs.

### 2.2 Clinicopathological testing

Upon admission, data including the breed, age, sex, comorbidities, clinical signs, medical history, and physical examination findings were recorded. Among the 53 data sets (with 20 clinically healthy cases and 33 suspected AP cases), the results of complete blood count (CBC) using IDEXX ProCyte Dx™ Hematology Analyzer (IDEXX Laboratories, Westbrook, MA, United States) along with blood smear examination and manual white blood cell differential counts were recorded. Serum biochemistry analysis included CRP measurement by BioNote, amylase and lipase by the IDEXX Catalyst One™ (IDEXX Laboratories, Westbrook, MA, United States), and creatinine, ALP, ALT, GGT, and glucose by AU480 Chemistry Analyzer (Beckman Coulter, United States). Serum CPL concentrations were determined by Vcheck CPL testing, and SNAP ® cPL tests were performed. The results of SNAP ® cPL were categorical, interpreted as either normal or abnormal by SNAPshot Dx Analyzer (IDEXX Laboratories, Westbrook, MA, United States). An abnormal SNAP ® cPL result is considered as having a CPL of ≥200 μg/L. Vcheck CPL provided quantitative results of serum CPL, with the detection range between 50 and 2000 μg/L. According to the manufacturer’s instructions, a Vcheck CPL result of ≥ 400 μg/L is consistent with pancreatitis, a result of 201–399 μg/L is considered to be equivocal for the diagnosis of pancreatitis, a result of <201 μg/L is considered unlikely to have pancreatitis. Eight out of 33 cases had abdominal ultrasonography examinations. Among 33 cases, 21 were hospitalized, and 11 had follow-up results of serum Vcheck CPL, serum CRP, and CBC on days 1, 2, and 3. Seven dogs have additional follow-up examinations on day 5. The decision to hospitalize and recheck these clinicopathological tests was made by their attending veterinarians. All clinical pathological analyses were performed at the Clinical Pathology Laboratory of the Veterinary Medicine Teaching Hospital of National Chung-Hsing University.

### 2.3 Consensus score calculation

A panel of 5 experienced small animal internists, with over 8 years of clinical practice, independently and retrospectively evaluated a total of 53 cases. Evaluation parameters included age, breed, clinical signs, medical history, physical examination findings, and laboratory evaluation (CBC, serum amylase, lipase, creatinine, ALP, ALT, GGT, and glucose). The internists assigned clinical scores of 0, 1, or 2 to each dog to assess the likelihood of AP. A score of “0” indicated exclusion of pancreatitis (not likely), “1” suggested an equivocal diagnosis that pancreatitis could not be ruled out based on the available information (likely), while “2” indicated a high suspicion of AP (very likely). Internists were blinded to the results of Vcheck CPL, SNAP ® cPL, and ultrasonography findings. When the assigned scores were the same from at least 3 internists, this consensus score was assigned to the case. No cases were excluded from the study because of failure to reach a consensus score.

### 2.4 Statistical analysis

All datasets underwent normality testing using the Shapiro–Wilk test. Comparisons of clinicopathological findings between dogs with AP and healthy dogs were conducted using Student’s t-test or Mann–Whitney U-test, as appropriate. Comparison of serum Vcheck CPL between three groups (healthy dogs, dogs with suspected AP with normal SNAP ® cPL results, and dogs with suspected AP with abnormal SNAP ® cPL results) was tested by the Kruskal-Wallis test followed by Dunn’s *post hoc* test. The correlation coefficient between Vcheck CPL and other clinicopathological variables was assessed using Spearman’s tests. A Vcheck CPL <50 and > 2000 was assigned a value of 49 and 2001, respectively. The association of the two diagnostic assays was compared by Fisher’s exact test and the Kappa coefficient was calculated to evaluate their agreement. Intraclass correlation coefficients (ICCs) were calculated to assess the agreement between clinical consensus score and the two respective diagnostic tests using the R package “irr” (12, 13), as well as agreement among the results of all assays. ICC represents a ratio of true variance over total variance (i.e., true variance plus error variance), indicating the extent to which measurements can be replicated. Thus, ICC reflects agreement among measurements on the same group of subjects. For the agreement testing among all assays, the consensus score and Vcheck CPL value were transformed into binary data. Consensus scores of 1 and 2 were regrouped as positive, while 0 was considered negative. An ICC value of <0.5 indicated poor agreement, 0.5–0.75 was considered moderate, 0.75–0.90 good, and > 0.90 excellent agreement (13). Unless specified, statistical analyses were otherwise performed using GraphPad Prism 9.0. The significance level was set at 0.05 across all statistics.

## 3 Results

### 3.1 Study population

Among the 33 dogs with suspected AP, 28 (85%) were purebred, comprising 10 Maltese, 4 Chihuahua, 4 Shiba Inu, 2 Dachshund, and 1 Beagle, Corgi, Golden Retriever, Labrador Retriever, Miniature Schnauzer, Spitz, Toy Poodle, and Yorkshire Terrier. The median age was 16 years (range: 2–20 years), with 33% being female and 67% male. The most common presenting complaints and clinical signs were anorexia or hyporexia (76%), followed by vomiting (45%), lethargy (36%), diarrhea (26%), and abdominal pain (15%).

Of the 20 healthy controls (HC), 10 (50%) were mixed-breed dogs, and 10 (50%) were purebred, including 2 Maltese, 2 Toy Poodle, and 1 of each Beagle, Chihuahua, Dachshund, German Shepherd, Golden Retriever, and Siberian Husky. The median age was 11.5 years (range: 6–20 years), with 40% being female and 60% male.

Out of the 8 cases that underwent abdominal ultrasonography examination, abnormalities in the pancreas were observed in only two dogs (25%). In both cases, hypoechogenicity was noted in the pancreatic parenchyma, accompanied by hyperechogenicity of the nearby adipose tissue. Additionally, one of the cases exhibited enlargement of nearby lymph nodes.

### 3.2 Serum Vcheck CPL, SNAP ® cPL, and clinical consensus score

**Figure 1:**
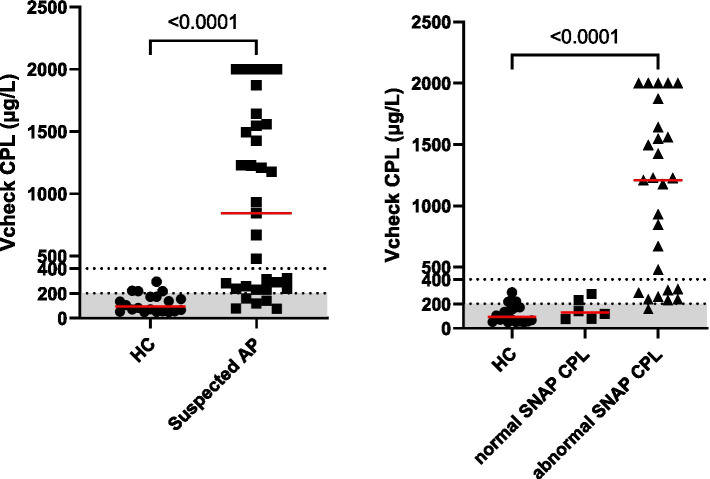
Serum Vcheck CPL concentrations in clinically healthy control dogs (HC, *n* = 20) and dogs with suspected pancreatitis (left panel). Further breakdown of dogs with suspected acute pancreatitis with normal SNAP ® cPL results (*n* = 6) or abnormal SNAP ® CPL results (*n* = 27) results are shown in the right panel. The gray area represents the reference interval.

Dogs with suspected acute pancreatitis had significantly higher serum Vcheck CPL (median: 843 μg/L, range: 77–2001, *p* < 0.0001, *n* = 33) than healthy control dogs (median: 94 μg/L, range: 49–294, *n* = 20), shown in Figure 1. Among dogs with suspected AP, 15% (5/33) had a serum Vcheck CPL of less than 200, 27% (9/33) between 200 and 400, and 58% (19/33) of above 400 (Figure 1). Among healthy dogs, 80% (16/20) had a serum Vcheck CPL of less than 200; while 20% had between 200 and 400. For SNAP ® cPL, among dogs with suspected AP, 82% (27/33) had a positive SNAP ® cPL, while 5% (1/20) of clinically healthy dogs had a positive SNAP ® cPL as well (Table 1). The agreement of the scoring of the five internists was good with an ICC of 0.77 (95% CI: 0.68–0.85, *p* < 0.001, *n* = 53). Among dogs with suspected AP, 70% (23/33) were assigned a clinical consensus score of 1, 24% (8/33) of 2, and 6% (2/33) of 0. All clinically healthy dogs were assigned a score of 0 from each internist; therefore, 100% of 0 for all. Two dogs suspected of AP received a consensus score of 0, indicating low likelihood, along with negative SNAP ® cPL results. Their serum Vcheck CPL concentrations were 78 and 118, respectively. Furthermore, all tested clinicopathological findings were within the normal range for both.

**Table 1:** Results of Vcheck CPL and SNAP ® cPL in the 53 dogs enrolled in this study.

Category	Vcheck CPL < 200 ng/mL unlikely	Vcheck CPL 200–400 ng/mL equivocal	Vcheck CPL > 400 ng/mL pancreatitis
SNAP ® cPL Normal	21	5	0
SNAP ® cPL Abnormal	1	7	19

We also regrouped the consensus score into positive (scores 1 and 2) and negative (score 0). The agreement of Vcheck CPL, SNAP ® CPL, and consensus score based on negative and positive was good with an ICC of 0.75 (95% CI: 0.64–0.84, *p* < 0.001, *n* = 53). The Vcheck CPL results are categorized into three groups based on the manufacturer’s product inserts: < 200, 200–400, and > 400 ng/mL. Meanwhile, SNAP ® cPL results are categorized into two groups: normal and abnormal.

Dogs with suspected AP with Vcheck CPL > 400 (*n* = 19), all had positive SNAP ® cPL and a consensus score of 1 or 2. We further recategorized Vcheck CPL results of dogs with suspected AP into positive and negative, and compared with the SNAP ® cPL results from the same dog. The positivity of Vcheck CPL is based on the cutoff value of 200 mg/L provided by the manufacturer’s instructions. The observed agreement between the Vcheck CPL and SNAP ® cPL was 89% with a Kappa of 0.77 (95% confidence interval: 0.61–0.94). Fisher’s exact test showed that Vcheck CPL and SNAP ® cPL were statistically associated (*p* < 0.0001, *n* = 53).

### 3.3 Daily monitoring of serum CPL and CRP

**Figure 2:**
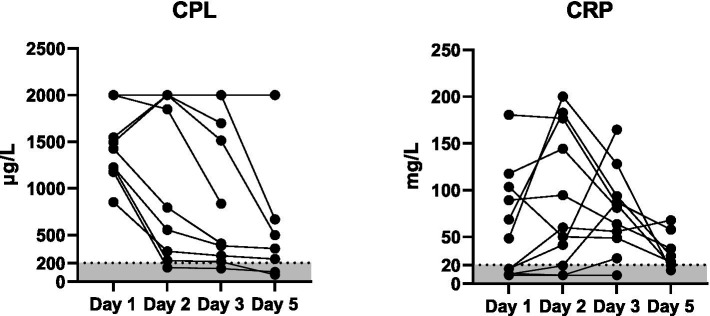
Serum concentrations of Vcheck CPL and CRP in 11 dogs with suspected acute pancreatitis on days 1 (*n* = 11), 2 (*n* = 11), 3 (*n* = 11), and 5 (*n* = 7). The gray area represents the reference interval, and the lines connect each case.

The changes in serum Vcheck CPL and CRP during hospitalization are illustrated in Figure 2. Upon admission (day 1), the median serum Vcheck CPL concentration was 1,495 μg/L (range: 853–2001, *n* = 11). There was no significant difference in serum CPL on day 2 (median: 1849 μg/L, range: 150–2001, *p* > 0.99, *n* = 11) compared to day 1. However, serum CPL decreased in 6 out of 11 dogs, with one dog returning to a normal range. By day 3, serum CPL significantly decreased (median: 837 μg/L, range: 141–2001, *n* = 11) compared to day 1. On day 5, the median CPL was 356 μg/L with a range of 74–2001 (*n* = 7). Serum CRP levels did not differ between days 1, 2, and 3 (*p* = 0.64). However, upon admission, 45% of dogs (5/11) had normal serum CRP concentrations (< 20 mg/L). Serum CRP concentrations became abnormal more frequently during hospitalization. On days 2, 3, and 5, 3 out of 11, 1 out of 11, and 2 out of 7 dogs, respectively, had CRP levels within the normal range.

### 3.4 Correlation between clinicopathological variables and Vcheck CPL values

**Figure 3:**
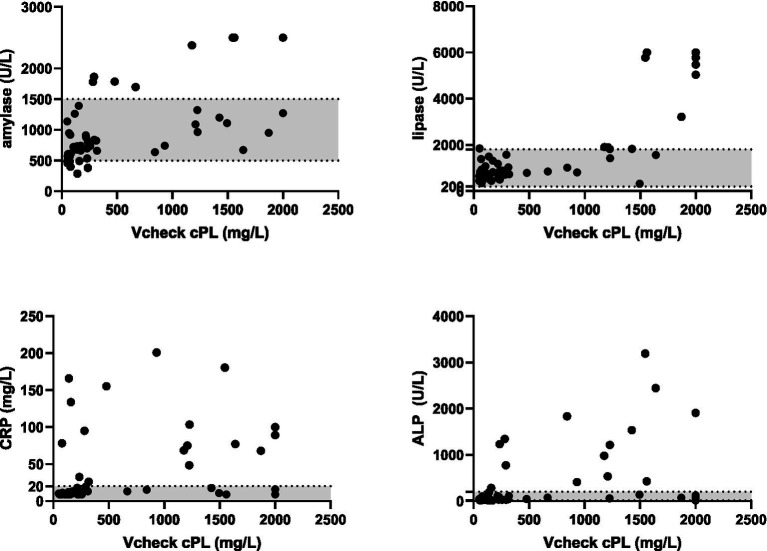
Correlation between serum amylase, lipase, CRP, and ALP concentrations with Vcheck CPL. Gray area represents the reference intervals.

Vcheck CPL values were significantly correlated with serum amylase (*r* = 0.65, *p* < 0.0001), lipase (*r* = 0.61, *p* < 0.0001), ALP (*r* = 0.60, *n* = 52, *p* < 0.0001), CRP (*r* = 0.48, *p* = 0.0003), and creatinine (*r* = 0.42, *p* < 0.0001), whereas Vcheck CPL did not significantly correlate with serum ALT (*p* = 0.27), GGT (*p* = 0.07), glucose (*p* = 0.10), and platelet count (*p* = 0.33). Additionally, Vcheck CPL values had a significantly negative correlation with hematocrit (HCT) (*r* = −0.49, *p* = 0.0002), RBC count (*r* = −0.44, *p* = 0.0009), hemoglobin (HGB) (*r* = −0.52, *p* < 0.0001), and lymphocyte count (*r* = −0.38, *p* = 0.0045). On the other hand, Vcheck CPL values had a significantly positive correlation with total WBC count (*r* = 0.45, *p* = 0.001), neutrophil count (*r* = 0.54, *p* < 0.0001), band cell appearance (*r* = 0.43, *p* = 0.0015), and monocyte count (*r* = 0.64, *p* < 0.0001) (Table 2; Figure 3).

**Table 2:** Significant correlation between blood test results and Vcheck cPL values.

Parameters	*r*	95% confidence interval	*p* value	*n*
Amylase	0.65	0.46–0.79	<0.0001	52
Lipase	0.61	0.40–0.76	<0.0001	52
CRP	0.48	0.24–0.67	0.0003	52
Creatinine	0.42	0.16–0.63	<0.0001	53
ALP	0.60	0.39–0.76	<0.0001	52
HCT	−0.49	−0.68– −0.25	0.0002	52
RBC count	−0.44	−0.64– −0.19	0.0009	53
HGB	−0.52	−0.70– −0.28	<0.0001	53
WBC count	0.45	0.19–0.65	0.001	52
Neutrophil count	0.54	0.30–0.71	<0.0001	53
Band cell appearance	0.43	0.17–0.63	0.0015	53
Monocyte count	0.64	0.44–0.78	<0.0001	53
Lymphocyte count	−0.38	−0.60– −0.12	0.0045	53

## 4 Discussion

The study revealed a significant correlation between a commercial point-of-care serum Vcheck CPL quantitative test and a semiquantitative SNAP ® cPL test, offering additional diagnostic support for canine acute pancreatitis (AP). In clinical practice, a comprehensive approach combining evaluation of the animal’s medical history, clinical symptoms, serum pancreatic lipase concentration, and, when feasible or necessary, abdominal ultrasonography, pancreatic cytology, or histopathology, is considered the most reliable for diagnosing pancreatitis. Serum pancreatic lipase immunoreactivity (PLI) is widely recognized as a diagnostic method for canine pancreatitis (4). Currently, multiple types of point-of-care serum PLI assays are available in the market, with Spec cPL showing the highest diagnostic performance in one study (14). However, the necessity of sending samples overseas to the United States for analysis in Southeast Asian countries (average 7 days turnover), combined with a cost exceeding three times that of SNAP ® cPL and 2.4 times that of Vcheck CPL (around USD 100 versus 32 and 42 dollars), has limited its widespread use. The precision of the Vcheck CPL assay was considered acceptable, with intra-, inter-, and total coefficients of variation being validated in Jakus’s study (15). The Vcheck CPL assay addresses this need for quantitative measurements by offering results that correlate well with the SNAP ® cPL test in cases of suspected acute pancreatitis. Furthermore, while providing numerical data offers degrees of differences, it might also aid in distinguishing between the equivocal and the true pancreatitis case. The correlation between Vcheck CPL and Spec cPL tests has been explored in various studies. Cridge et al. reported poor repeatability for Vcheck CPL results (14), while Kim et al. observed a strong correlation between these tests, highlighting the potential applicability of Vcheck sPL in clinical setting (16). These findings underscore the necessity for further research to validate the performance, reliability, and clinical utility of these diagnostic methods in diverse contexts. In addition, advancements in diagnostic tools such as the Catalyst Pancreatic Lipase Test on the Catalyst platform are becoming increasingly accessible worldwide. These innovations emphasize the importance of quantitative measurements in diagnosing pancreatitis; however, further research is essential to assess the comparative effectiveness of platforms among Vcheck, Speck cPL and Catalyst.

Notably, most dogs suspected of having AP received a clinical score of 1, suggesting the internists cannot rule in or rule out AP when blinded with Vcheck CPL results. This indicates the importance of this additional testing to aid in diagnosis. Among dogs with suspected AP, four received a score of 1, but their Vcheck CPL was less than 200 as well as a negative SNAP ® cPL test. Their CBC, serum amylase, lipase, ALP, ALT, and GGT levels were within normal reference intervals. Two out of the four dogs exhibited abnormal serum CRP concentrations, one had increased serum creatinine, and the remaining dog showed normal results for all blood parameters tested, suggesting that extensive blood work is necessary to diagnose AP and rule out other conditions. Two dogs with a consensus score of 1 had a negative SNAP ® cPL and a Vcheck CPL in the equivocal range. One had all tested variables within the reference range, while the other exhibited abnormal liver panels and serum CRP levels, indicating the presence of other diseases. These dogs with results in discrepancy might be explained by truly without AP or delayed testing. Studies have found that serum lipase activity rapidly decreased in dogs with AP as fast as in 1 day (8). Two dogs with all negative results from three tests were most likely excluded from AP, but the Vcheck CPL testing was requested due to the need for the exclusion of AP. Collectively, this emphasizes the necessity of specific CPL testing to aid internists in better excluding and including potential differentials. Despite employing rapid screening methods, potential false negative results may still occur in dogs with pancreatitis, particularly in cases of acute-on-chronic disease with significant pancreatic tissue loss (17). Similarly, false positive results can arise in dogs without clinically significant acute pancreatitis due to conditions like intestinal foreign body, obstruction, or peritonitis. Pancreatic inflammation may also be secondary to duodenal reflux, ischemia, or generalized peritonitis associated with conditions such as septic peritonitis, abdominal hemorrhage, or intestinal foreign bodies. Furthermore, some underlying diseases such as metabolic or endocrinologic diseases especially in aged animals cannot be ruled out. Therefore, despite severe histological and ultrasonographic evidence of pancreatic inflammation, pancreatitis may not always be the primary cause of clinical signs in dogs. Limited use of abdominal imaging and pancreatic ultrasound in our study has added complexity to accurate diagnostics by internists as it has shown to be positively correlated with AP (18). Nevertheless, these potential complex clinical scenarios provided a valuable platform for real-world assay comparisons, enhancing the relevance and applicability of our findings.

The utilization of serum CPL levels could be beneficial in managing canine AP in certain circumstances. This phenomenon is similar to the findings of Keany et al., who suggested that serum cPLI and possibly CRP could serve as objective biomarkers for clinical changes in hospitalized dogs with acute pancreatitis (19). In this study, the notable decrease in CPL by day 3 suggests a potential trend of treatment response or disease resolution. Previous studies have shown a rapid decline in both CPL and CRP levels within 1 day post-treatment (8). However, quantitative CPL measurement could reveal persistently abnormal CPL levels despite clinical remission. This disparity may be linked to the hospitalization of more severe cases, potentially indicating the progression to chronic pancreatitis or other pancreatic disorders such as neoplasia or edema. Variations exist in some cases, similar to the previous study (20). While the reduction in serum cPL immunoreactivity (cPLI) concentration is associated with enhanced clinical outcomes in dogs hospitalized with acute pancreatitis, the utilization of serial serum cPLI as an objective biomarker for monitoring clinical alterations in hospitalized dogs with acute pancreatitis necessitates further investigation.

In our study, we observed moderate positive correlations between Vcheck CPL and SNAP ® CPL values and serum amylase, lipase, ALP, and creatinine. Serum amylase and lipase levels can increase due to pancreatic inflammation, but may also originate from various tissues, leading to their relatively low specificity as markers for pancreatitis (20, 21). The positive correlation with ALP may be attributed to reactive hepatopathy and extrahepatic bile duct obstruction resulting from pancreas inflammation, given the proximity of the extrahepatic biliary system to the pancreatic head (22). The moderate positive correlation between Vcheck CPL results and creatinine might be associated with pancreatitis in three distinct circumstances: renal failure preceding and potentially precipitating pancreatitis, pancreatitis complicated by renal failure, and the co-occurrence of renal failure and pancreatitis in patients with other systemic diseases (23).

Vcheck CPL and SNAP ®cPL values were significantly correlated with blood cell parameters, showing positive correlation with many white blood cell-related parameters including WBC count, neutrophil count, band cell appearance, and monocyte count, and negative correlation with red blood cell-related parameters such as RBC count, HGB, and HCT. As pancreatitis is an inflammatory disorder, neutrophils, and monocytes are recruited to the pancreas during the early stage of acute pancreatitis, followed by dendritic cells, mast cells, and T cells (24, 25). Additionally, the body produces an excessive number of neutrophils and induces programmed cell death of lymphocytes in response to inflammation, as indicated by the negative correlation between Vcheck CPL results and lymphocyte count. Recent data suggest that markers of systemic inflammation, such as the neutrophil/lymphocyte ratio, which reflects immunological instability, can predict the prognosis of various diseases, including acute pancreatitis. A higher ratio is associated with a worse prognosis (26–28) in humans. Pancreatitis can also lead to anemia, primarily due to inflammation, with gastrointestinal bleeding being a less common cause. In Cai et al., human patients with acute pancreatitis and anemia exhibited increased disease severity, a higher incidence of acute kidney injury, and a poorer prognosis compared to those without anemia (29). While in human studies, a stronger correlation in numerical values is observed, limited literature in the canine domain suggests a prevalence of around 55–61% exhibiting higher white blood cell and neutrophil counts among dogs with pancreatitis, approximately 9% showing elevated monocyte counts, and anemia being present in 28% of cases (30).

Several limitations need to be addressed in our study. Firstly, we did not assess serum CPL concentrations using another quantitative test due to its high cost. Ideally, to ensure accuracy, samples should be measured by two machines with the same methodology. However, since the manufacturer provided in-house data demonstrating a high correlation with the other quantitative test, we alternatively utilized SNAP® cPL, a semi-quantitative test, to evaluate the correlation between the two tests. This approach limited comparison with samples in the equivocal range. Nevertheless, dogs with Vcheck CPL > 400 consistently exhibited abnormal SNAP ® cPL results, aligning with their clinical signs. Secondly, as the data were collected at a teaching hospital, the cases included in the study may have been more severe or had comorbidities compared to those in primary veterinary clinics, potentially biasing the blood test results. Moreover, histological examinations were not performed on these dogs. Lastly, as only severe cases were monitored, follow-up samples were not available for all dogs in the study, which might bias the results and prevent correlation evaluation between different diagnostic results over time.

In conclusion, our study observed a moderate to strong agreement between Vcheck CPL and SNAP ® cPL, highlighting its potential clinical utility in the quantification of CPL. This commercially available point-of-care quantitative testing provides rapid results with only 25 μL of serum samples needed. Future study on comparisons to the quantitative spec PLI would be needed to substantiate comparable uses of Vcheck CPL in diagnosis of AP. It is important to acknowledge that the primary purpose of these pancreatic assays is to promptly rule out pancreatitis rather than serve as definitive diagnostic tools. Based on our study results, Vcheck CPL may emerge as a convenient and affordable assay for quantifying serum pancreatic lipase levels in dogs, particularly in regions with limited access to other quantitative lipase assays.

## Data Availability

The original contributions presented in the study are included in the article/supplementary material, further inquiries can be directed to the corresponding author/s.
